# CARE NEEDS AND LIFE SATISFACTION IN FRAIL OLDER ADULTS: THE MODERATING EFFECT OF AGE-FRIENDLY ENVIRONMENTS

**DOI:** 10.1093/geroni/igae098.2932

**Published:** 2024-12-31

**Authors:** Inhye Jung, Miseon Kang, Seolah Lee, Hyo Jung Lee

**Affiliations:** Yonsei University, Seoul, Republic of Korea; Yonsei University, Seoul, Republic of Korea; Yonsei University, Seoul, Republic of Korea; Yonsei University, Seoul, Republic of Korea

## Abstract

Frail older adults often face challenges in performing Activities of Daily Living (ADL) and Instrumental Activities of Daily Living (IADL), leading to diminished quality of life. Age-friendly environments, designed to facilitate aging in place, aim to enhance the well-being of older adults and their family members of all ages by modifying their surroundings and promoting adaptation. This study investigates how age-friendly environments moderate the association between care needs and life satisfaction among frail older adults, elaborating on their role in the process. Using data from the 2020 National Survey of Older Koreans, we identified 1,435 frail respondents. Being frail was defined according to the scores summed with three questionaries asking respondents’ physical abilities, which were based on the Study of Osteoporotic Fracture criteria. Age-friendly environments were categorized into Physical, Service, and Sociocultural domains following WHO principles. Multi-regression analysis, employing the Process Macro Model 4, revealed that age-friendly service and sociocultural environments mitigate the negative impact of care needs on life satisfaction among frail older adults. These findings underscore the potential of age-friendly environments to improve the quality of life and support aging in place for frail older adults. Creating supportive environments for older adults with frailty is essential for promoting the overall well-being of these older adults and their care providers.

